# Treatment of osteoarthritis of the knee with a topical diclofenac solution: a randomised controlled, 6-week trial [ISRCTN53366886]

**DOI:** 10.1186/1471-2474-6-44

**Published:** 2005-08-08

**Authors:** Philip A Baer, Lisa M Thomas, Zev Shainhouse

**Affiliations:** 1 Malvern Medical Centre, Toronto, Canada; 2 Clinical Research, Dimethaid Research Inc., Markham, Canada

## Abstract

**Background:**

Topical NSAIDs have been proven to relieve the symptoms of osteoarthritis (OA) in short-term studies (2 weeks). To justify its chronic use, efficacy of a topical NSAID over a longer term of study should be demonstrated. The efficacy and safety of a topical diclofenac solution over a 6-week treatment course in symptomatic primary OA of the knee was investigated.

**Methods:**

216 men and women, age 40–85 years, with radiologically confirmed primary OA of the knee and a flare of pain at baseline following discontinuation of prior therapy were enrolled into this double-blind study. Participants applied either a topical diclofenac solution (Pennsaid^®^) or vehicle control solution (carrier with no diclofenac); 40 drops 4 times daily directly to the painful knee(s), without massage, for 6 weeks. Pre-planned primary efficacy outcome measures included the core continuous variables pain relief and improved physical function measured by the Western Ontario and McMaster Universities (WOMAC) LK3.1 OA Index, and improved patient global assessment (PGA). Secondary efficacy measure was reduced stiffness. Safety assessments included adverse events and vital signs.

**Results:**

The topical diclofenac group had a significantly greater mean change in score (final minus baseline) compared to the vehicle control group for pain (-5.2 *vs*. -3.3, p = 0.003), physical function (-13.4 *vs*. -6.9, p = 0.001), PGA (-1.3 *vs*. -0.7, p = 0.0001) and stiffness (-1.8 *vs*. -0.9, p = 0.002). The mean difference between treatment arms (95% confidence interval [CI]) was 1.9 (0.7 to 3.2), 6.5 (2.5 to 10.5), 0.6 (0.2 to 0.9), and 0.9 (0.3 to 1.4), respectively. Safety analyses showed that topical diclofenac caused skin irritation, mostly minor local skin dryness, in 42/107 (39%), leading to discontinuation of treatment in 5/107 (5%) participants.

**Conclusion:**

This topical diclofenac solution demonstrated relief at 6 weeks of the symptoms of primary osteoarthritis of the knee.

## Background

Meta-analysis of previous trials of topical non-steroidal anti-inflammatory drugs (NSAIDs) concluded that they effectively treat the pain of acute soft tissue injuries [1] and chronic musculoskeletal conditions [2,3]. Current evidence-based recommendations for the management of osteoarthritis (OA) support the use of topical NSAIDs and rubefacients [4-6] as a therapeutic option potentially with fewer gastrointestinal risks than oral NSAIDs [7]. However, a recent critical meta-analysis concluded that claims of pain relief in OA by currently available topical NSAIDs are supported by only a limited number of randomised controlled trials of small size and brief duration, with no data demonstrating efficacy beyond 2 weeks [8].

In this report, we present the efficacy and safety results from a 6-week controlled trial using a newer topical diclofenac solution in knee OA. Effect size data and number-needed-to-treat (NNT) are presented, facilitating comparison with the previously reviewed data.

## Methods

### Participants and inclusion/exclusion criteria

This study was conducted from November 1999 to August 2000, at 17 medical centres across central Canada, following approval by a central ethics review board (Integrated Research Incorporated, Ethics Review Committee, Montreal, QC). Participants were recruited from the physician's private practice or the surrounding community. At the screening visit, after providing written, informed consent, each participant underwent a screening interview and was eligible to proceed to washout if all inclusion criteria and no exclusion criteria were met.

Inclusion criteria specified men and non-pregnant women, age 40–85 years, with primary OA of at least one knee, and a flare of pain after withdrawal of prior therapy with either an oral NSAID or acetaminophen (used at least 3 days per week during the previous month). Primary OA was defined by deterioration and abrasion of articular cartilage (joint space narrowing) or formation of new bone (osteophytes) at the joint surface of the knee (medial tibio-femoral, lateral tibio-femoral or patello-femoral), demonstrated on a radiological examination carried out within the previous 3 months [9]. Pain was measured by the Western Ontario and McMaster Universities LK3.1 OA Index (WOMAC) 5-item pain subscale, each item scored on a 5-point Likert scale (none = 0; mild = 1; moderate = 2; severe = 3; extreme = 4) [10]. Pain was scored at the screening visit, following which prior therapy was withdrawn. The patient scored the pain again at the baseline visit. A flare was defined as an increase in total pain subscale score of at least 2 and at least 25%, with a baseline total pain score of at least 6 (out of a possible 20), and a score of ≥2 (out of a possible 4) on at least one of the 5 items in the WOMAC pain subscale.

Participants were excluded if they had secondary arthritis related to systemic inflammatory arthritis (including rheumatoid arthritis, psoriatic arthritis, post-infectious arthritis and metabolic arthritis, traumatic arthritis or surgical joint replacement); corticosteroid use: (a) oral corticosteroid within the previous 14 days, or (b) intramuscular corticosteroid within 30 days, or (c) intra-articular corticosteroid into the study knee within 90 days, or (d) intra-articular corticosteroid into any other joint within 30 days, or (e) topical corticosteroid at the site of application within 14 days; intra-articular viscosupplementation (e.g., Synvisc^®^) into the study knee in the preceding 90 days; ongoing use of prohibited medication including NSAID, other oral analgesic, muscle relaxant, or low-dose antidepressant for any chronic pain management; ongoing use of glucosamine or chondroitin (unless used continuously for 90 days prior to study entry); sensitivity to diclofenac, acetylsalicylic acid (ASA) or any other NSAID, acetaminophen, dimethyl sulphoxide, propylene glycol, glycerine or ethanol; clinically-active renal, hepatic or peptic ulcer disease; history of alcohol or drug abuse; lactation; concomitant skin disease at the application site; current application for disability benefits on the basis of knee osteoarthritis; fibromyalgia; other painful or disabling condition affecting the knee; or participation in another investigational drug trial in the previous 30 days.

### Interventions

At the baseline visit, all patients that met the final entry criterion of a flare of pain were randomly assigned to receive one of two treatments: (a) topical diclofenac solution (Pennsaid^®^; Dimethaid Research Inc.), consisting of 1.5% (w/w) diclofenac sodium in a patented carrier containing dimethyl sulphoxide (45.5%, w/w), propylene glycol, glycerine, ethanol and water, or (b) vehicle control solution, consisting of the complete carrier (including dimethyl sulphoxide, 45.5% w/w) but no diclofenac. Participants applied a dose of 40 drops of study solution (about 1.3 mL) to the affected knee 4 times daily for up to 6 weeks. The participant was instructed to apply 10 drops of solution to each side of the knee (front, back, medial and lateral) either dripped directly onto the knee or first into the hand, and then spread over the site without massage. Compliance was verified by weighing the solution bottles at each visit. If the other knee was painful at any time during the study, it was treated and evaluated for safety, but efficacy analysis was performed on only the study knee – the one with the greater baseline pain score (or the dominant knee if both had the same score). Consumption of acetaminophen (up to four 325-mg tablets per day) was permitted for residual knee or other body pain throughout the treatment period, but not during the washout period prior to baseline assessment or during the week prior to final assessment at week 6. ASA (≤ 325 mg/day) was permitted for cardiovascular prophylaxis.

### Outcome measures

The primary outcome measures were defined as the change from baseline to final assessment of the study knee in the 3 core continuous variables [11] pain and physical function, assessed using the WOMAC subscales, and patient global assessment (PGA). There was no intermediate assessment of efficacy. The WOMAC is a validated questionnaire [12] consisting of 24 questions (5 on pain, 17 on physical function and 2 on stiffness), each scored on a 5-point Likert scale (see Participants). The PGA question asked: "How has the osteoarthritis in your study joint been over the last 48 hours?" and was scored on a Likert scale (very good = 0; good = 1; fair = 2; poor = 3; very poor = 4). This question focuses on the treated site, unlike a PGA in an oral NSAID trial that can probe the non-signal joints. Secondary measure was change in stiffness. Ancillary measures defined *a posteriori *were pain on walking – the first question of the WOMAC pain subscale (referred to as 'use-related pain' [13]) – and the following dichotomous variables: 50% improvement in pain [3]; final PGA score of "good" or "very good" [3]; and response based on OMERACT-OARSI responder criteria [14] (a responder is defined as a participant with ≥ 50% improvement in pain or function that was ≥ 20% of the scale, or ≥ 20% improvement in at least two of pain, function or PGA that was ≥ 10% of the scale).

### Safety analyses

Safety was assessed during all clinic visits (weeks 3 and 6) and telephone 'visits' (weeks 1 and 5). Safety variables included adverse events, application-site dermatological reactions and vital signs. Adverse events were identified using open-ended questions and a checklist covering common oral NSAID side effects. Dermatological assessment of the knee was based on a standard scale [15] and any abnormality was recorded as an adverse event. All adverse events were categorised according to Coding Symbols for Thesaurus of Adverse Reaction Terms (COSTART) [16]. Laboratory assessment was not done.

### Sample size

Based on a power of 80% and a Type I error rate of α = 0.05_2-tailed_, a sample size of 80 participants per group was required to detect an estimated important difference of 2 between the treatment arms, in the change in WOMAC pain dimension score from baseline to final (with standard deviation of 4.5). A total sample size of 200 participants (100 per treatment group) was specified in the protocol, which allowed for a non-evaluable rate of up to 20%.

### Randomisation and blinding

The study kits were prepared, labelled and numbered according to a computer-generated randomisation schedule created by an outside consultant using a randomly chosen block size of 4 or 6. They were shipped to the sites in multiples of complete blocks to ensure that a balanced number of participants was assigned to the two treatment arms within each site. As a participant qualified for study entry at the baseline visit, the investigator assigned him/her the next randomisation number in a sequential manner. The randomisation schedule was concealed from the investigators, their support staff, study participants and the sponsor's clinical research personnel, until final data lock. Except for the individual participant identification number on the label, the two study solutions were identical clear, colourless liquids packaged in opaque bottles.

### Statistical analysis

Safety analyses were performed on all randomised participants who received at least one dose of study solution. There was no imputation of missing safety data. Efficacy analyses were performed on an intent-to-treat (ITT) group, defined as a subset of all randomised participants who met critical inclusion criteria (primary OA by history, an abnormal radiological study, and any degree of knee pain), as per ICH guidelines [17]. For any missing efficacy data in the ITT analysis, the last observation was carried forward. A per-protocol group was defined based on stricter adherence to study conduct, including requirement for a moderate flare of knee pain (see Participants) and treatment continuing for at least 40 days.

Baseline demographic and clinical variables were analysed by Chi-square or Student's t-Test. Adverse event incidence was analysed by Chi-square or Fisher's Exact Test. Continuous variables (WOMAC dimensions, PGA and pain on walking) were analysed by ANCOVA with baseline score as the covariate without adjustment for testing secondary/alternative objectives. The dichotomous variables were analysed by Chi-square test. All statistical tests were two-sided and were performed at the 0.05 level of significance.

## Results

### Participant flow

**Figure 1:**
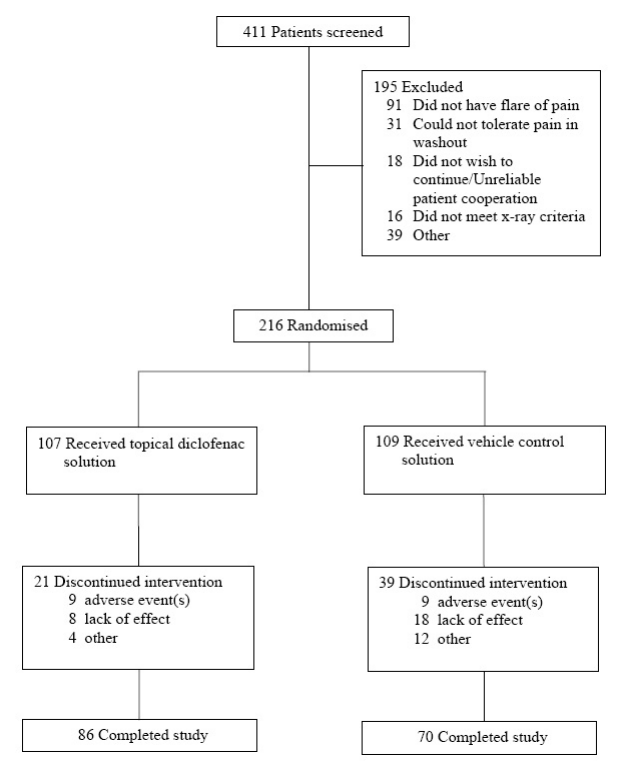
Flow of participants.

Two hundred and sixteen participants were randomised to treatment with either topical diclofenac (n = 107) or vehicle control (n = 109). All participants received their allocated intervention. More participants in the topical diclofenac group (86 [80%]) completed the entire 6-week treatment period compared to the vehicle control group (70 [64%]; p = 0.008). Discontinuation rate due to an adverse event was similar in both groups. Dropout due to lack of effect was lower for topical diclofenac (8 [7.5%]) compared to vehicle control (18 [16.5%]; p = 0.041). No participant was lost to follow-up (Fig. 1).

### Baseline demographic and clinical characteristics

No significant difference was found between treatment groups in baseline demographic and clinical characteristics (Table 1). The mean (SD) screening and baseline pain scores were 8.2 (2.7) and 13.0 (3.2) in the topical diclofenac group versus 8.3 (3.0) and 12.8 (3.1) in the vehicle control group (12.9 [3.2] overall). Most participants treated both knees, either from baseline or by the end of the trial.

**Table 1:** Baseline demographic and clinical characteristics of treatment groups

	**Topical diclofenac (n = 107)**	**Vehicle control (n = 109)**
Age (years)	65.0 (11.0)	64.6 (10.9)
Women, number (%)	56 (52.3)	66 (60.6)
Race/ethnicity, number (%)		
White	88 (82.2)	91 (83.5)
Black	8 (7.5)	3 (2.8)
Oriental	3 (2.8)	2 (1.8)
Other	8 (7.5)	13 (11.9)
Weight (kg)	89.9 (18.1)	86.5 (17.3)
Height (m)	1.65 (0.11)	1.65 (0.10)
Heart rate (bpm)	74.1 (10.0)	74.3 (9.1)
Systolic blood pressure (mm Hg)	137.6 (16.3)	133.6 (15.6)
Diastolic blood pressure (mm Hg)	81.4 (9.1)	79.7 (8.7)
Total x-ray score*	7.7 (5.4)	7.0 (5.0)
Screening pain score	8.2 (2.7)	8.3 (3.0)
Baseline^† ^pain score	13.0 (3.2)	12.8 (3.1)
Baseline^† ^physical function score	40.7 (11.9)	40.4 (11.2)
Baseline^† ^stiffness score	5.2 (1.5)	5.2 (1.5)
Patient global assessment score‡	3.1 (0.8)	3.2 (0.8)
Participants treating two knees at baseline, number (%)	64 (59.8)	70 (64.2)
Participants treating two knees at final, number (%)	84 (78.5)	89 (81.7)

Data are presented as mean (SD) unless otherwise indicated.

*Total score of joint space narrowing, marginal osteophytes formation and subchondrial sclerosis for each knee compartment (medial, lateral, patello-femoral); maximum score possible was 27.

†After washout of prior therapy; pain scale ranged from 0 (no pain) to 20 (extreme pain); physical function scale ranged from 0 (no difficulty) to 68 (extreme difficulty); stiffness scale ranged from 0 (no stiffness) to 8 (extreme stiffness).

‡Patient global assessment was measured using a Likert scale, ranging from 0 (very good) to 4 (very poor).

Mean (SD) duration of treatment in the topical diclofenac group was 38.2 (9.9) days versus 34.4 (12.5) days in the vehicle control group (p = 0.013). Compliance with the dosing regime was 83.1 % and 84.5% for the topical diclofenac and vehicle control groups, respectively. No significant difference was noted in the mean (SD) consumption of rescue acetaminophen tablets per day between the topical diclofenac (0.9 [0.9]) and vehicle control groups (1.1 [1.0]; p = 0.079).

### Efficacy analyses

Four of 216 randomized participants were not included in the ITT analysis group because they violated major entry criteria: 2 participants lacked radiological confirmation of OA (no radiological examination for one participant and a normal examination for the other), and 2 participants had secondary OA (related to osteochondroma). Inclusion of these participants yielded the same results in a subsequent re-analysis (data not shown).

#### Planned analyses

There was a significantly greater improvement in score with topical diclofenac compared to vehicle control (Table 2) for pain (-5.2 vs. -3.3; p = 0.003,), physical function (-13.4 vs. -6.9; p = 0.001), PGA (-1.3 vs. -0.7; p = 0.0001) and stiffness (-1.8 vs. -0. 9; p = 0.002) Analysis of the per protocol group of 128 participants confirmed the statistical superiority of topical diclofenac over vehicle control for the primary and secondary outcome measures (p < 0.01; data not shown).

**Table 2:** Efficacy evaluation of the continuous variables

**Efficacy variable**	**Treatment group**	**N**	**Baseline score, mean (SD)**	**Change in score mean (SD)**	**Mean difference in change (95% CI)**	**P-value**	**Effect size (95% CI)**
Pain	Topical diclofenac	105	13.0 (3.1)	-5.2 (5.0)	1.9 (0.7 to 3.2)	0.003	0.41 (0.14 to 0.68)
	Vehicle control	107	12.7 (3.2)	-3.3 (4.3)			
Physical function	Topical diclofenac	105	40.9 (11.9)	-13.4 (16.3)	6.5 (2.5 to 10.5)	0.001	0.44 (0.16 to 0.71)
	Vehicle control	107	40.3 (11.3)	-6.9 (13.2)			
Patient global assessment	Topical diclofenac	105	3.1 (0.8)	-1.3 (1.3)	0.6 (0.2 to 0.9)	0.0001	0.47 (0.19 to 0.74)
	Vehicle control	107	3.2 (0.7)	-0.7 (1.1)			
Stiffness	Topical diclofenac	105	5.3 (1.4)	-1.8 (2.1)	0.9 (0.3 to 1.4)	0.002	0.43 (0.15 to 0.70)
	Vehicle control	107	5.2 (1.5)	-0.9 (2.0)			
Pain on walking	Topical diclofenac	105	2.7 (0.8)	-1.2 (1.2)	0.4 (0.1 to 0.7)	0.014	0.34 (0.07 to 0.61)
	Vehicle control	107	2.7 (0.8)	-0.8 (1.1)			

#### A posteriori analyses

There was a significantly greater improvement in score with topical diclofenac compared to vehicle control (Table 2) for pain on walking (-1.2 vs. -0.8; p = 0.014). The response rate for at least a 50% reduction in pain (Table 3) was significantly greater following topical diclofenac treatment compared to vehicle control (46/105 [43.8%] vs. 27/107 [25.2%]; p = 0.004). The topical diclofenac group had a significantly greater number of participants with good or very good PGA response (43.8% vs. 16.8%; p <0.0001) compared to the vehicle control group and of OMERACT-OARSI responders (65.7% vs. 49.5%; p = 0.017).

**Table 3:** Efficacy evaluation of the dichotomous variables

**Efficacy variables**	**Treatment group**	**N**	**Number (%) of participants**	**p-value**	**Number-needed-to-treat (95% CI)**
50% reduction in pain	Topical diclofenac	105	46 (43.8)	0.004	5 (3–17)
	Vehicle control	107	27 (25.2)		
OMERACT-OARSI responder*	Topical diclofenac	105	69 (65.7)	0.017	6 (3–33)
	Vehicle control	107	53 (49.5)		
Good or very good PGA response	Topical diclofenac	105	46 (43.8)	<0.0001	4 (3–7)
	Vehicle control	107	18 (16.8)		

*A responder is defined as a participant with ≥ 50% improvement in pain or function that was ≥ 20% of the scale, or ≥ 20% improvement in at least two of pain, function or patient global assessment that was ≥ 10% of the scale.

### Adverse events

The major adverse effect reported was dry skin at the application site, occurring in 42/107 (39.3%) and 23/109 (21.1%; p = 0.004) of topical diclofenac and vehicle control participants, respectively (Table 4). A skin-related adverse event led to discontinuation of only 5 participants in the topical diclofenac group. All skin reactions resolved promptly upon withdrawal of treatment. Abdominal pain and dyspepsia each were reported in 4 [3.7%] participants in the topical diclofenac group compared to 1 [0.9%] participant in the vehicle control group, but this difference was not significant (p = 0.21).

**Table 4:** Number (%) of adverse events

**Adverse Event**	**Topical diclofenac (n = 107)**	**Vehicle control (n = 109)**
Gastrointestinal reaction		

Abdominal pain	4 (3.7)	1 (0.9)
Constipation	1 (0.9)	1 (0.9)
Diarrhea	1 (0.9)	0
Dyspepsia	4 (3.7)	1 (0.9)
Gastritis	1 (0.9)	0
Melena	0	1 (0.9)
Nausea	1 (0.9)	2 (1.8)

Application-site skin reaction		

Dry skin	42 (39.3)*	23 (21.1)
Rash	2 (1.9)	4 (3.7)
Paresthesia	2 (1.9)	2 (1.8)
Pruritus	0	2 (1.8)

Other reaction		

Headache	6 (5.6)	10 (9.2)
Halitosis	2 (1.9)	0
Taste Perversion	4 (3.7)	2 (1.8)

*p < 0.01 vs. vehicle control

## Discussion

Published guidelines have incorporated topical NSAIDs as recommended treatment for OA of the knee [4-6]. However, there has been controversy surrounding the adequacy of data supporting their benefit beyond 2 weeks [2,3,8,18]. Moreover, the studies identified in these meta-analyses generally did not conform to current standards for OA trial design [11,19]. In contrast, the present trial utilized standardized radiological and clinical entry criteria and measured efficacy with validated outcome measures. Baseline pain score was substantial; mean (SD) score was 12.9 (3.2) out of a maximum of 20, indicating a flare of pain following withdrawal of prior therapy. Analysis of all of the primary and secondary measures demonstrated that treatment with this topical diclofenac solution relieved the symptoms of primary knee OA at 6 weeks in this study population. Two other recently published trials using this topical diclofenac solution showed it to be superior to vehicle control and/or placebo; a 4-week, non-flare trial of 248 participants [20] and a 12-week, flare trial of 326 participants [21]. As with most NSAID trials, the subject population in this study was selected by the inclusion criterion of a flare of pain, which demonstrates the potential to respond to NSAID/analgesic. In clinical practice, an individual not taking an analgesic may have considered previous NSAID therapy ineffective, in which case s/he would not be expected to respond to topical diclofenac. However, where an individual is intolerant to oral NSAID, one may consider topical diclofenac as a treatment option.

Comparison of efficacy results from independent trials with various treatments is facilitated by the introduction of benchmark determinants that are mathematically derived from the experimental raw data, such as effect size [22] for improvement of a continuous variable (e.g. "How much did the patient's pain improve, relative to placebo?"). We calculated an effect size (95% CI) of 0.41 (0.14 to 0.68) for pain relief, 0.44 (0.16 to 0.71) for improved physical function and 0.34–0.47 for improved measures of PGA, stiffness and pain on walking (Table 2). In contrast, Lin et al. [8] calculated a pooled effect size for pain relief of 0.04 (essentially no effect) in 3 placebo-controlled topical NSAID trials of 4 weeks duration. A meta-analysis of 23 oral NSAID trials for OA knee, lasting 2–13 weeks, reported a pooled effect size of 0.32 for pain reduction and 0.29 for improving physical function [23]. Another meta-analysis of 14 OA trials found a pooled effect size of 0.37 for pain reduction with oral NSAIDs and 0.44 for coxibs [24]. Zhang et al. [25], using data from 2 oral NSAID studies of 6–12 weeks duration, calculated a pooled effect size for OA pain reduction of 0.34.

Efficacy of a treatment is being expressed increasingly as a dichotomous result, e.g. "Did the patient's pain improve by 50%; yes or no?". We derived the response rate for each dichotomous variable from our raw data, and demonstrated the superiority of topical diclofenac over vehicle control for 50% reduction in pain, achieving a good or very good final PGA response, and 'response' by OMERACT-OARSI criteria (Table 3). The benchmark determinant for comparing dichotomous efficacy results of various treatments is the number-needed-to-treat (NNT) [26]. We calculated a NNT between 4 and 6, depending upon the variable (Table 3). In their meta-analysis of topical NSAIDs, Mason et al. [3] cited 5 placebo-controlled trials of short duration for OA knee pain – 8 days (1 trial), 14 days (3 trials), and 28 days (1 trial). Their definition of clinical success, representing approximately a 50% reduction of pain, was estimated using patient or physician global assessment as the outcome measure (4 trials and 1 trial, respectively). They calculated a NNT of 5.3.

Few oral NSAID studies have reported dichotomous data. Osiri et al. [26] reported a NNT for pain improvement of 4.4 with etodolac and 3.8 with tenoxicam. Defining improvement as an increase of at least 2 grades (on a 0–5 scale) in the patient's global rating of arthritis, Edwards et al. [27] reported a NNT of 11–13 for valdecoxib treatment of OA.

The OMERACT-OARSI initiative used a consensus approach to derive dichotomous 'responder' criteria [14]. Through their vast meta-analysis of suitable trials, the authors found that for trials of oral NSAIDs vs. placebo the responder rates were 65.4% and 45.9% respectively. Responder rates of 60–65% have been reported for 13-week treatment of OA with celecoxib and lumiracoxib, with placebo responder rates of 49–53% [28,29]. The OMERACT-OARSI initiative did not look at topicals but we applied its criteria to this study and found a responder rate for topical diclofenac of 65.7% with a placebo responder rate of 49.5%, similar to their oral NSAID data.

A caveat in the application of the mathematical benchmarks, effect size and NNT, is the influence of trial design, outcome measures and patient population on the apparent magnitude of response to a given treatment. Because the trials with topical diclofenac were designed according to the OARSI guidelines, like most recent NSAID and cyclooxygenase-2 (COX-2) inhibitor studies, such comparison of results is reasonable [19]. Although the data observed for topical diclofenac in this trial are comparable to other NSAID trials, a direct head-to-head comparison trial is required to prove equivalency of two treatments. A previously published 12-week comparative trial of 622 participants with OA knee confirmed the clinical equivalence between topical diclofenac solution and oral diclofenac [30].

Safety analysis revealed no serious clinical adverse effects and only minor application-site skin reactions, mostly skin dryness, following treatment with topical diclofenac. While dimethyl sulphoxide in the carrier acts as a penetrant [31], it also dissolves normal surface oils and leaves the skin dry. Common skin lubricants may prevent most application site reactions and any related discontinued therapy, but such products were not permitted in this trial in order to detect the maximum potential side effect profile of the study solutions. The low dropout rate due to skin reactions (5/107 [4.7%] for topical diclofenac) suggests patient acceptance of the overall topical treatment regime.

The use of a checklist to prompt the patient about possible adverse events likely yielded a high estimate of the true incidence of gastrointestinal adverse reactions caused by topical diclofenac. The report of abdominal pain and dyspepsia each in 3.7% of patients is consistent with what was seen in other published trials of this topical diclofenac [20,21] and much lower than commonly experienced with oral NSAIDs or COX-2s [30]. Those other trials included results of laboratory testing and found minor abnormality of liver enzymes in 2–5%, creatinine in 1% and haemoglobin in 2% of patients, significantly lower than with oral diclofenac [30]. This safety profile can be predicted from the low systemic availability of topically applied diclofenac. Although the patient applies a daily dose (40 drops, 4 times a day) of 86 mg of diclofenac to the knee, the blood level is only 12 ng/mL [31]. The level reported after oral administration of 50 mg Voltaren^® ^is 1500 ng/mL [32]. Similar improved safety with topical NSAIDs has been reported previously [33].

## Conclusion

Topical diclofenac solution provides 6-week relief of the symptoms of knee OA. The data in this and previous reports provide substantial evidence for the efficacy and safety of topical diclofenac solution in chronic OA.

## Competing interests

LMT and ZS are employees of Dimethaid Research Inc.

PAB was a principal investigator in the trial, and was remunerated for his participation.

## Authors' contributions

PAB was a major investigator in the trial and was involved in data interpretation. LMT was involved in analysis and interpretation of the data and writing of the manuscript. ZS was involved in trial design and conduct, data review and writing of the manuscript. All authors reviewed and approved the final draft of the manuscript.

## Pre-publication history

The pre-publication history for this paper can be accessed here:


